# How Tupanvirus Degrades the Ribosomal RNA of Its Amoebal Host? The Ribonuclease T2 Track

**DOI:** 10.3389/fmicb.2020.01691

**Published:** 2020-07-28

**Authors:** Clara Rolland, Bernard La Scola, Anthony Levasseur

**Affiliations:** ^1^Aix-Marseille Université, UMR MEPHI (Microbes, Evolution, Phylogeny and Infections), IRD, APHM, Faculté de Médecine, Marseille, France; ^2^IHU Méditerranée Infection, Marseille, France; ^3^Institut Universitaire de France, Paris, France

**Keywords:** giant virus, tupanvirus, rRNA shutdown, ribonuclease T2, amoeba

## Abstract

Tupanviruses are giant viruses recently discovered in Brazil from extreme environments: *Tupanvirus soda lake* (TPV-SL) and *Tupanvirus deep ocean* (TPV-DO). Unexpected features in Tupanviruses is the cytotoxic effect observed during infection, where the virus degrades the ribosomal RNA (rRNA) of its amoebal host. Interestingly, only TPV-SL causes this rRNA shutdown. We performed a genomic comparison of the two strains to determine potential modifications explaining the absence of rRNA degradation by TPV-DO. Whole genome comparisons were performed as well as more in-depth analysis at the gene level. We also calculated selective pressure on the orthologous genes between the two viruses. Our computational and evolutionary investigations revealed a potential target: a ribonuclease T2. These enzymes are known to be involved in cellular RNA catabolism such as in lysosomal degradation of rRNA. Our results suggest a functional ribonuclease localized in acid compartment closely related to ribonuclease T2 from eukaryotes. Silencing of the RNAse T2 gene of TPV-SL abolished its rRNA shutdown ability thereby correlating *in silico* assumption to the experimental evidence. In conclusion, all our results pointed to RNAse T2 as a target for explaining the difference for rRNA degradation ability between both strains.

## Introduction

The giant viruses revolutionized the field of virology. Their isolation redefined the structural morphology and the size of the virion abolishing the small size parameter of viruses (inferior to 200 nm) (La Scola et al., 2003; Boyer et al., 2009; Reteno et al., 2015; Andreani et al., 2016; Levasseur et al., 2016). The length and composition of their genome show an unsuspected complexity in the viral world (La Scola et al., 2003; Philippe et al., 2013). Indeed, a genome mosaicism has been observed with many genes with different origins (bacteria, eukaryote or archaea) (Colson et al., 2018). Finally, the presence of elements from translational machinery, the boundary between viruses and intracellular parasites, constitutes a new stage in this redefinition (Schulz et al., 2017; Bajrai et al., 2019).

Tupanvirus is one of the most complex giant viruses discovered at this time. Two strains: *Tupanvirus soda lake* (TPV-SL) and *Tupanvirus deep ocean* (TPV-DO), were isolated respectively from soda lake and marine sediments at a depth of 3000 m (Abrahão et al., 2018). These two strains compose the “Tupanvirus” genus proposed by Rodrigues et al. (2018). One of the features of Tupanviruses is that they have an almost complete translation apparatus with 20 aminoacyl tRNA synthetase (aaRS), 70 tRNA and many other factors associated with the transcription or maturation of mRNA. Despite this exceptional viral translation apparatus, a crucial factor is still lacking: ribosomal proteins, essential in translation process. The tupanvirus’ infection is characterized by the formation of bunches, aggregate of infected amoebae with uninfected cells. This is the first time this way of increasing viral progeny has been observed in a giant viral infection (Oliveira et al., 2019). Furthermore, it is also the first virus to infect a broad range of hosts, such as *Tetrahymena* sp., *Acanthamoeba castellanii neff*, *Vermamoeba vermiformis*, or *Willaertia magna* (Abrahão et al., 2018; Silva et al., 2019). With this panel of host, different types of infections were identified including a cytotoxic infection at high multiplicity of infection (MOI 100) without replication. Only the TPV-SL strain is able to induce this cytotoxic effect characterized by a severe shutdown of the ribosomal RNA (rRNA) of the host. Several cellular pathways described in eukaryotes illustrate the rRNA degradation for the control of a proper translation, cell homeostasis or in response to environmental stresses. Ribosomes are the target that leads to rRNA degradation by selective or non-selective pathways such as RNautophagy, macroautophagy, or ribophagy (Kazibwe et al., 2019). It has been demonstrated that the phenomenon is not related to autophagy/ribophagy mechanism (Abrahão et al., 2018). Other mechanisms that could explain this rRNA degradation are the implication of ribonuclease (exo or endoribonuclease) such as RNase I, RNase R, or PNPase in bacteria (Deutscher, 2009). In literature, three other viruses are known to induce rRNA degradation in association with ribonuclease. *Hepatitis A virus* HAV cp strain (HM175/18f clone) and *Human coxsackievirus* B1 (CVB1) in FrhK4 cells are capable to degrade rRNA, and authors supposed a replicative advantage (Kulka et al., 2003). The third virus described to degrade rRNA is the baculovirus *Autographa californica* multiple nucleopolyhedrovirus (AcMNVP) in BM-N cells (*Bombyx mori* permissive cells to nucleopolyhedrovirus). For authors, an antiviral mechanism of cells would be involved (Hamajima et al., 2013). Interestingly, the strain TPV-DO did not lead to the cytotoxic profile during an infection, thereby giving us a unique opportunity to understand the difference between the two strains to clarify the rRNA shutdown’s underlying mechanism.

The objective of this work is to carry out a depth comparative analyse of genomes of the two viruses to identify potential modifications explaining the absence of rRNA degradation with TPV-DO. For this purpose, a comparison of the two strains by genomic (structural and functional) and evolutionary approaches have been set up to test several hypotheses. Analyses were performed at the genomic level, at two different stages. The first step of exploration was the “macroscopic level” of genome, the viewable level, where macro mutations can be visible with this question: did a genomic rearrangement occur? The second stage focused on the discrepancies in the genome, i.e., in the coding part: Was there a gene loss or gain between the two viruses? The two different environments where the viruses were discovered might play a role in their evolution, hence the hypothesis: have selective pressures on specific genes been modified? Therefore, evolutionary shift was studied between orthologous genes in order to detect functional shift associated with the shutdown of the rRNA of the host.

## Materials and Methods

### Genome Comparison

The genomes of TPV-SL and TPV-DO were retrieved from NCBI GenBank database. Alignment and comparison of the two genomes was performed by MAUVE software (v2.4.0) and with CONTIGuator software online (Darling et al., 2004; Galardini et al., 2011).

### Genes Homology

Gene prediction was computed using Prodigal software (v2.6.3) (Hyatt et al., 2010). The predicted proteins with a size less than 50 amino acids were discarded and 25 genes between 50 and 99 amino acids analyzed with Phyre2 software as having an abnormal tri-dimensional structure were eliminated of the dataset (Kelley et al., 2015).

Homology between the two sets of predicted gene sequence was first investigated using ProteinOrtho software (v5.16b) with default parameters (e-value cut-off of 1.0e-5; identity threshold of 25% and coverage threshold of 50%) (Lechner et al., 2011). Then, a complementary analysis was achieved using the Blast software suite (Blastp, Blastn, and tBlastn) on predicted proteins to refine results (Altschul et al., 1990; Camacho et al., 2009).

Identification of conserved domains of predicted protein unique to either viruses was realized using in combination CD-search tool online, ProDom software online (v2012.1) and Phyre2 software online (Servant et al., 2002; Kelley et al., 2015; Marchler-Bauer et al., 2017).

SignalP v4.1 and PSORT online software were used to identify a cellular localization of interesting sequences (Nakai et al., 1999; Nielsen, 2017).

The structure of interesting sequences was analyzed by multiple alignment. A prediction of 3D structure was made with Phyre2 software online and visualized with BIOVIA Discovery Studio Visualizer 2019 software (Kelley et al., 2015; Dassault Systèmes Biovia, 2017).

### Phylogeny of the Targeted RNAse T2 Sequence

First, when a target sequence was found, the phylogeny was performed using BLASTP against the non-redundant database with default parameters. All sequences identified by BLAST were retrieved, such as sequences originate from different amoeba species and already studied in the literature corresponding to the target sequence. We aligned the sequences with MUSCLE on MEGA 7 software and a phylogenetic tree was made using the Maximum Likelihood method (ML) with JTT as substitution matrix with 1000 bootstraps (Kumar et al., 2016).

### Selective Pressure

A dN/dS ratio was calculated to evaluate the selective pressure applied on orthologous genes between the two viral strains. The genes with the highest dN/dS ratio were selected and analyzed as previously done with Blast and CD-search (Altschul et al., 1990; Marchler-Bauer et al., 2017).

### Evolutionary Analyses

Evolutionary analyses were performed as previously described (Levasseur et al., 2006, 2010). Briefly, protein and DNA sequences were retrieved from the National Center for Biotechnology Information. Protein sequences were aligned using MUSCLE (Edgar, 2004). Correspondence between protein alignment and each DNA sequence was established using Wise2 software followed by manual adjustments (Birney et al., 2004).

The codeml program of the PAML (Phylogenetic Analysis by Maximum Likelihood) software package was applied to test for positive selection (Yang, 1997). PAML uses a maximum likelihood algorithm to assign likelihood scores to different models for selection. If a model incorporating positive selection gave a higher likelihood score than a null model without positive selection, this constitutes evidence for positive selection. Model A implemented by Yang & Nielsen was used (Yang et al., 2000). This model enables ω (= dN/dS) to vary both between sites and between lineages, and has been implemented within the maximum likelihood framework. This model was then used to construct likelihood ratio tests (LRTs) by comparison against models that do not identify positive selection. The null hypothesis is the site model M1a, which assumes two site classes with 0 < ω0 < 1 and ω1 = 1 for all branches (Yang et al., 2005; Zhang et al., 2005).

### Tupanvirus RNAse T2 Silencing

#### Viral Production and Titration

Tupanvirus strain soda lake was co-cultured with *A. castellanii* strain Neff in peptone-yeast extract glucose (PYG) medium. Cell culture flasks containing 7 × 10^6^ *A. castellanii* were infected with tupanvirus at an multiplicity of infection (M.O.I) of 0.1 and incubated at 32°C. To purify the virus, the co-culture was centrifugated at low speed (1,700 × *g* per 10 min) and the supernatant was filtered across a 0.8 μm membrane to remove residual amoebas. Then supernatant was washed three time in Page’s modified Neff’s amoeba saline (PAS) by high centrifugation (10,000 × *g* per 10 min) to pellet the virus. After viral purification, virus titer was determined by end-point dilution (Reed and Muench, 1938).

### Silencing of RNAse T2

For gene silencing, we targeted the RNAse T2 using small interfering RNA (siRNA). Two systems were synthesized by Eurogentec (Liège, Belgium), the duplex siRNA_161 with sense (5′-UGCAUGGUAUUUGGCCUGAAUAUUA-3′) and the anti -sense (5′-UAAUAUUCAGGCCAAAUACCAUGCA-3′) and the duplex siRNA_325 with sense (5′-CAUGGUACUUGUGCCU CAACUGAUU-3′) and the anti-sense (5′-AAUCAGUUGAGG CACAAGUACCAUG-3′). We diluted 600pmol of duplex siRNA and 50 μl of Lipofectamine RNAiMAX (Invitrogen) in 200 μl of PYG according to the manufacturer’s recommendations. Before transfection, 5 × 10^5^ fresh *A. castellanii* were put onto small flask (12.5cm2) with 2ml of PYG during 20 min to allow them time to adhere. After this, the siRNA-Lipofectamine solution were added to flasks containing amoebas and incubated at room temperature for 10 min. Then TPV-SL at a multiplicity of infection of 100 were added to flasks. This time point was defined as H0. At 9h post-infection, the cells were collected by low centrifugation (1,700g per 10min) and subjected to RNA extraction according to the manufacturer’s instructions (Qiagen RNeasy Mini Kit, Hilden, Germany). From the extracted RNA, 10 μL of each sample was electrophoresed in 1.5% agarose gel and run at 135V for 20 min.

## Results

### Structural Annotation of Genomes of TPV

**FIGURE 1:**
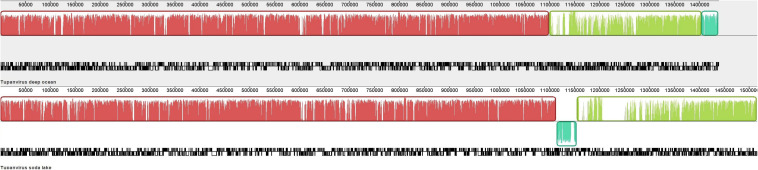
Whole genome alignment of Tupanviruses by MAUVE software (Darling et al., 2004). The locally collinear blocks (LCB) are colored and the coding density are in black/white under the LCB.

In order to detect putative macro mutation at the genome scale, the genome architecture of both TPV was investigated (Figure 1). An inversion of the terminal of TPV-SL’s genome was observed in comparison with TPV-DO. This change preceded a region with a lack of similarity positioned in the starting point of TPV-DO’s genome (Supplementary Figure 1).

Comparison of genome co-linearity of Tupanviruses showed a structure with three blocks of similarities (Locally Collinear Blocks, LCB). Indeed, the length of the three LCBs for TPV-DO are 1,099,109 bases (from position 1 to 1,099,109) for the first block. For the second, the length is 305,119 bases (b) meaning from 1,099,110 to 1,404,229 and the third block represents 33,849b (from position 1,404,230 to 1,438,079). For TPV-SL, the first is composed of 1,113,694b, the second of 40,029b (from 1,113,695 to 1,153,724) and the third LCB length is of 361,145b (from position 1,153,725 to 1,514,870). Minor differences emerged from this comparison, in particular a discrepancy indicating the lack of similarity between the two viruses at positions 1,205,690 to 1,250,939 in the genome of TPV-DO. The density of genes was not influenced despite a lower similarity in specific location, where a lack of correspondence was observed such as for the gap previously cited.

### Gain and Loss of Genes in Both TPV

**FIGURE 2:**
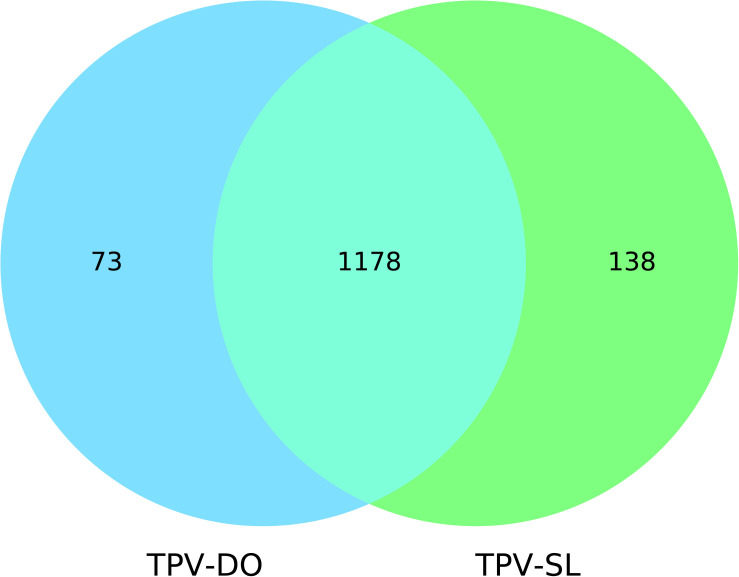
Venn diagram of genes shared and not shared by the two Tupanviruses.

Genes prediction resulted in 1251 and 1339 predicted genes for TPV-DO and TPV-SL, respectively. A total of 1178 clusters of orthologous genes (COGs) were detected between both strains. 73 and 138 genes were unique in TPV-DO and TPV-SL, respectively (Figure 2). Those unique genes are distributed along all both genomes through the three LCB described overhead. In the small block in which occurs inversion, one gene was found for TPV-DO (TPV-DO_1250) and two for TPV-SL (TPV-SL_1008 and TPV-SL_1011). TPV-SL also possess 23 paralogous genes, all identified as hypothetical protein or putative orfan.

The 73 putative proteins unique of TPV-DO were analyzed and the results indicate that 38 are ORFans, 19 are annotated as hypothetical protein and 16 had a hit in NCBI non-redundant database (Supplementary Table 1). To go further, a search of known functional and conserved domains revealed that for 49 proteins without Blast identification, no domains were identified. On the other hand, for 23 others, at least one domain was found. For examples, a serine hydrolase domain was recovered for TPV-DO_952 gene, which contains the domains β lactamase and FliW (Flagellar assembly factor) or such as TPV-DO_971 where, a C-term E3 ligase domain was identified (domain associated as a virulence factor in *Escherichia coli*). Functional annotation of these domains was also confirmed by structural prediction.

Concerning TPV-SL, the same analyses were performed on TPV-SL predicted genes (Supplementary Table 1). Among these 138 proteins, TPV-SL_330 (AUL79063.1) represents a potential target. Indeed, the predicted function is a ribonuclease function belonging to the ribonuclease T2 (RNase T2 family) (Table 1).

**TABLE 1:** Predicted function of TPV-SL_330 (AUL79063.1) sequence identified as RNase T2.

Software	Accession	Name	Position	Score	e-value
*Blastp*	ARF11526.1	T2 family ribonuclease	1–209	164	2e-47
*CD-Search*	cd01061	RNAse_T2_euk	23–210	167.51	1.35e-52
*ProDom*	#PD001112	Ribonuclease T2	24–212	322	8e-34

	**Template**	**Name**	**Position**	**Confidence (%)**	**Id (%)**

*Phyre2*	c3t0oA	Ribonuclease T2	23–212	100	28

**FIGURE 3:**
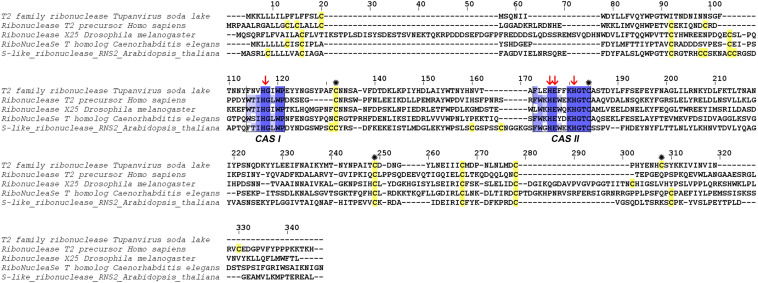
Alignment of RNase T2 from *Tupanvirus soda lake* with reference eukaryote sequences. The two conserved CAS I and CAS II regions, characteristic of RNase T2 enzyme, are marked and conserved amino acids enlighten in blue (the intensity of color increase with the degree of conservation). Catalytic histidines are pointed with red arrows and cysteines are in yellow with those important for the active site marked with an asterisk.

**FIGURE 4:**
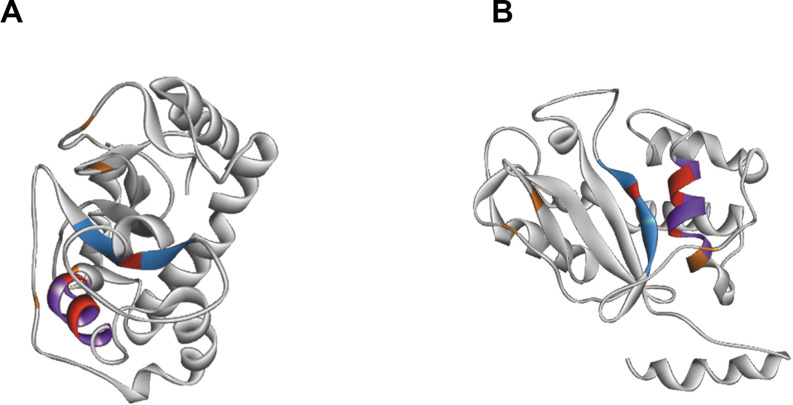
Structure of RNAse T2 from *Tupanvirus Soda Lake*. Panels **(A,B)** shows the RNase T2 with two different views. The active site is marked by CAS I domain in blue and CAS II domain in purple. The important amino acids of the active site are showed in red and stabilizing cysteines in orange.

At the structural level, sequences alignment shows that two sites of conserved amino acids (CAS I and CAS II) are conserved, with slight changes, in comparison to consensus sequences (Figure 3). In the first conserved block (8 amino acids), an asparagine (position 2), a valine (position 3) and an isoleucine (position 6) were retrieved, instead of a threonine, leucine or isoleucine and a leucine respectively in comparison with sequence already deeply studied from *Homo sapiens* or *Drosophila melanogaster*. In the second site (12 amino acids), a leucine in position 2 and a lysine in position 6 replace a tryptophan. Three histidines (His55, His104, His109) and glutamic acid (Glu105) important for the enzyme are identified in the active site. In the sequence, 7 cysteines were also detected, especially the coupled cysteine 2, 3 (in position 70 and 113 of the RNAse) and cysteine 4, 7 (respectively in position 176 and 205) important for the stabilization of the active site by establishing disulfide bridge (Figure 4).

Beyond structural predictions, the protein subcellular localization was studied. The online tools SignalP and PSORT, used in combination, allowed the prediction of a signal peptide in N terminal position (from the first amino acids to the 18th) and a putative localization in compartment with acid pH, such as lysosome or vacuoles.

#### Phylogeny

**FIGURE 5:**
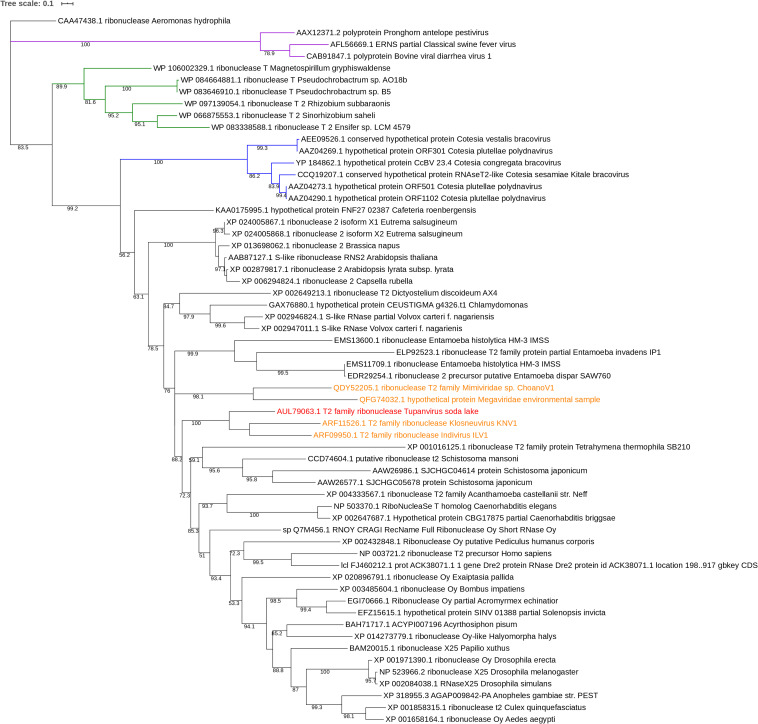
Phylogenetic tree of RNase T2 sequences. The analysis was performed using Maximum Likelihood method (ML) with a JTT substitution matrix in 1000 replicates. Bootstraps below 50 were discarded. The branch labeled in purple corresponds to RNA viruses (pestiviruses), the one in blue to DNA viruses and the one in green to alphaproteobacteria. RNase T2 sequence of TPV-DO is highlighted in red and those of giant viruses in orange.

Phylogenetic tree was performed using a maximum likelihood framework (Figure 5). It was rooted by ribonuclease sequence of the gammaproteobacteria *Aeromonas hydrophila.* The base of the tree is formed of the *A. hydrophila* ribonuclease sequence, a sister branch composed of RNA viruses (Pestivirus) and a third branch containing the ramifications of the rest of phylogeny. This last branch contains a branching with a cluster corresponding to *Alphaproteobacteria* sequences and a second branch with, in one hand, a cluster of DNA viruses, and, on the other hand, all eukaryotes sequences of ribonuclease including giant virus sequences. Two giant viruses *Mimiviridae sp. ChoanoV1* and *Megaviridae environmental sample* form a unique branch, sister to the one corresponding to *Entamoeba* species. The closest relatives or the RNase T2 sequence of TPV-SL are *Klosneuvirus* and *Indivirus*, giant viruses retrieved from metagenomic analyses. They constitute one branch and the closest eukaryote organisms are *Tetrahymena thermophila* and *Schistosoma* species (*Schistosoma mansoni* and *Schistosoma japonicum*).

**FIGURE 6:**
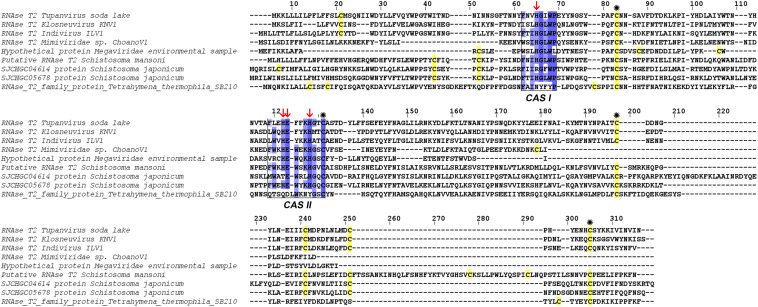
Alignment of RNase T2 sequences from phylogeny. The alignment shows TPV-SLRNase T2 sequence compared with sequences of all other giant viruses and closest eukaryotes in phylogeny. CAS I and CAS II regions, characteristic of RNase T2 enzyme, are marked and conserved amino acids enlighten in blue (the intensity of color increase with the degree of conservation). Catalytic histidines are pointed with red arrows and cysteines stained in yellow with those important for the active site marked with an asterisk.

The comparison of RNase T2 conserved domains in the branch of Tupanvirus exhibit 75% of amino acid similarity between TPV-SL and Klosneuvirus for CAS I domain (similarity corresponding to identical amino acid and amino acid with the same properties). For the CAS II domain, only 67% of similarity is found. Between TPV-SL and Indivirus, the similarity is estimated to 88% for CAS I and 58% for CAS II. Concerning the catalytic sites, three histidines and the glutamic acid, all are conserved as for the four cysteines in order to stabilize the active site (Figure 6). If the analysis is expanded to the closest branch containing eukaryotes (*T. thermophila* and *Schistosoma* species), results show 38% of similarity between Tupanvirus and *Tetrahymena hydrophila* for CAS I and 25% for CAS II without conservation of amino acids involved in catalysis, thus highlighting a possible inactive enzyme. However, cysteines are maintained. The data for *Schistosoma* sp. indicate a better conservation with a similarity of 88% for CAS I domain and 58% for CAS II for *Schistosoma mansonii.* We observe the same percentage of similarity for CAS I for *S. japonicum* and 75% for CAS II. Both organisms possess the four amino acids for catalysis and the four cysteines involved in stabilization of the active site.

### RNAse T2 Implication in rRNA Degradation *in vivo*

**FIGURE 7:**
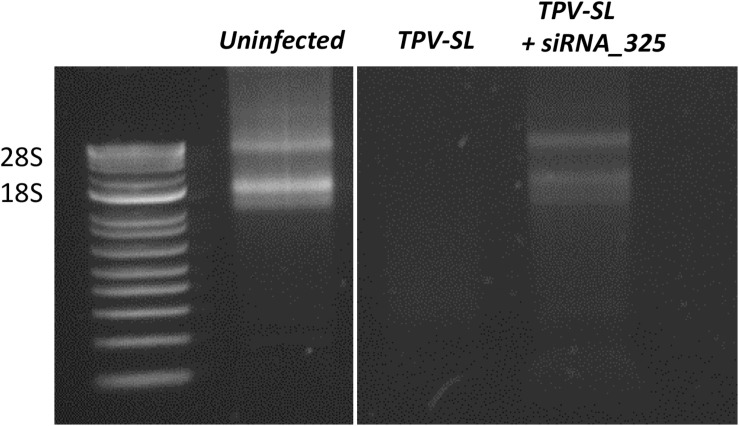
Gene silencing of the RNAse T2 and effect on the rRNA shutdown induced by Tupanvirus infection. Electrophoresis gel showing ribosomal 18S and 28S RNA from *A. castellanii* under three conditions: uninfected cells, infected cells with *Tupanvirus soda lake* (TPV-SL) at MOI 100 and infected cells by TPV-SL and siRNA_325.

In order to test our hypothesis about the implication of the RNAse T2 in the host rRNA degradation, silencing of the RNAse T2 gene were performed using siRNA targeting two different areas of the gene. Two duplexes of siRNA were tested (i.e., siRNA_161 and siRNA_325). The siRNA_161 did not prevent the rRNA degradation induced during TPV-SL infection. Conversely, the siRNA_325 allowed the restauration of rRNA 18S and 28S in amoeba (Figure 7).

### Selective Pressure

In order to understand the selective pressure applied on genes shared by both strains, a dN/dS was calculated. Only genes with a dN/dS ratio higher than 0.2 were selected. The selection yielded 25 genes with a ratio included between 0.21 and 99 (Supplementary Table 2).

The results indicate that, among the 25 selected genes, only 4 already have a function assigned. The protein TPV-SL_1023 possess, a DNA topoisomerase II function, TPV-SL_1137 is a transposase, TPV-SL_1138 is a resolvase, and TPV-SL_1279 is a double, homebox protein 4-like. In addition of these proteins, two others firstly identified as hypothetical proteins possess conserved functional domains. A NADAR domain (*E. coli* swarming mobility protein YbiA) for TPV-SL_730 and, two domains for TPV-SL_1136: Zinc finger RING-type and Sel-1 repeat.

## Discussion

In this work, we were interested in the degradation mechanism of rRNA induced by Tupanvirus during host infection. The discrepancy between both strains, in term of rRNA shutdown effect, offers a unique opportunity to screen for potential differences between both strains.

The first step in our analysis consisted of determining whether a genomic rearrangement occurs in either or both Tupanviruses, which may explain the observed difference in functional mechanism. Our results showed a very close genomic structure between the two strains.

The second step focused on the gain or loss of genes between the two viruses. Horizontal gene transfer is a common evolutionary event and giant viruses are able to gain or loose a number of genes. Among the specific genes of TPV-SL, most of them are hypothetical proteins without associated functions a feature of giant viruses (Colson et al., 2017). Interestingly, one of them: TPV-SL_330 (AUL79063.1) protein, was annotated as a RNase T2. It is a ribonuclease belonging to the 2′–3′ cyclizing RNase family, present in most of organisms (fungi, bacteria, virus, eukaryotes) without specificity of cleavage for a DNA base (Luhtala and Parker, 2010). The most interesting feature remains its acid pH functioning in correlation with the acidification of amoebae’s cytoplasm observed during a Tupanvirus infection (Abrahão et al., 2018). In literature, this class of enzymes was well-described, showing a structure having two highly conserved domains of amino acids in all organisms (CAS I and CAS II), which constitute the active site (Irie, 1999; Luhtala and Parker, 2010). These domains are kept at TPV-SL with slight modifications of the amino acids, which mainly retain the same biochemical properties, e.g., in position 3 a valine instead of a leucine in CAS I, both being non-polar and aliphatic amino acids.

For catalysis, four amino acids are critical for RNase T2 activity: three histidines and one glutamic acid. In addition, cysteine residues ensuring the stability of the active site are also strongly preserved. The study of the structure of TPV-SL’s T2 RNase, and its comparison with others already described clearly demonstrated a strict and conserved positioning of these essential amino acids. Additional analyses supported these results by confirming the presence of an active histidine site. In conclusion, the functional annotation of this protein converges to functional assignment as RNase T2 type. Furthermore, the presence of a signal peptide at the beginning of the sequence and the prediction of a localization of the enzyme indicates an address signal to acid pH compartments such as the lysosome. Knowing that infection with Tupanvirus causes ribosome sequestration in large vesicles (Abrahão et al., 2018), it can assume that TPV-SL T2 RNase, could be exported to these vesicles. Considering that T2 RNase is exported when the virus has infected its host, it can be assumed that this is a general mechanism, since Tupanvirus also induces this degradation of rRNA in other protists, particularly in *Tetrahymena* sp. (Abrahão et al., 2018). This degradation is accompanied by a reduction in the physiological activity of the protist as well as its phagocytosis capacity. Thus, the mechanism of rRNA degradation might maximize the ability of the virus to find a host favorable to its multiplication. Although productive viral replication is not strictly required for cytopathology, Tupanvirus appears to be an unusual exception of pathogenic virus without viral replication requirement among the nucleocytoplasmic large DNA viruses (NCLDV).

Some evidences in other organisms indicate that RNase T2 enzymes are also involved in phosphate and/or nucleotides recycling to maintain cell homeostasis especially under stress conditions (Hillwig et al., 2011; MacIntosh, 2011). An alternative hypothesis to explain the rRNA degradation might be that TPV-SL induces a stress for cells. This condition leads to segregation of ribosomes into vacuoles used by the virus to retrieve nucleic acids.

In order to study the origin and distribution of RNase T2 in giant viruses, a phylogenetic study was performed. This study suggests that TPV-SL’s RNase T2 is linked to other giant viruses reconstituted *in silico*: Klosneuvirus and Indivirus related to the Mimiviridae family (Schulz et al., 2017). Both viruses exhibit an active site with amino acids essential for catalysis (histidines and glutamic acid) and for the stabilization of the site (two coupled cysteines). Only two others giant viruses possess this enzyme, but with a lower sequence similarity than TPV-SL. All those viruses are comprised in the extended Mimiviridae family, but no evidence of spreading in giant viruses came to light for now. An independent acquisition for the five giant viruses identified in this analysis is still questioning. The presence of RNase T2 in several viruses from the extended Mimiviridae family suggests a functional involvement of the enzyme leading to selective constraint in favor of gene conservation. One could question about the absence of RNase T2 gene in TPV-DO despite its phylogenetic proximity with TPV-SL. According to the Red Queen hypothesis, microorganisms are submitted to perpetual evolution to constantly adapt and proliferate in their environment (Van Valen, 1973). Isolation of TPV-DO and TPV-SL from such different environments (soda lake and marine sediments at a depth of 3000 m) could be a driving force to explain the gain and loss disequilibrium for both viruses. The closest eukaryotes are *Schistosoma* sp. and *T. thermophila.* Despite a well-known infection of *T. thermophila* by TPV-SL, the low degree of similarity seems to indicate that TPV-SL RNase T2, does not derive from this organism. However, the position of TPV-SL’s sequence within eukaryote sequences can suggest an acquisition of gene from an eukaryote organism, probably during infection of Tupanvirus. A second hypothesis can be a possible evolution from a common ancestor between giant viruses and eukaryotes (Guglielmini et al., 2019).

In order to confirm the implication of the RNAse T2 of TPV-SL in the host rRNA degradation, silencing experiments were performed using siRNA. Two siRNA (siRNA_161 and siRNA_325) were designed and tested in gene silencing experiments. The siRNA_161 showed no effect on rRNA shutdown and did not inhibit the RNAse T2 of TPV-SL. In contrast, the siRNA_325 exhibited a recovery of the host ribosomal 18S and 28S transcripts. Interestingly, we noted variable efficiency of the gene silencing using siRNA_325 with different bands intensity of 18S and 28S transcripts following recovery. This variability could be due to parameters such as conditions of growth and infection of the amoeba, molecular extraction and triggering of the silencing machinery. Moreover, the partial recovery of rRNA could also suggest the involvement of other proteins in addition to the RNAse T2, especially in this virus where a majority of proteins are still ORFans without functional characterization. In order to avoid all variabilities depending on the specific targeted area of transcripts or on silencing efficiency, gene knockout experiments of the RNAse T2 gene of TPV-SL will be recommended as an alternative and stable technique. In conclusion, *in vivo* silencing experiments are strongly encouraging to support the involvement of RNAse T2 in the rRNA degradation.

Finally, the evolution of the two Tupanviruses was analyzed. The analysis of selective pressures points that 25 genes evolved under different constraints between the two virus strains with relaxed selective pressure. The RNase T2 is not included in this list and results do not highlight other potential targets for the explanation of amoeba rRNA degradation by TPV-SL.

This work focused on a comparative genomic approach. However, transcriptomic comparison of the two strains could also be considered for explaining the rRNA shutdown’s difference. Actually, fine regulations at the level of promoters could also be at the origin of transcriptomic differences, thus explaining the loss of degradation of rRNAs at TPV-DO.

According to these computational and experimental results, RNAse T2 represents a target of choice for explaining the difference for rRNA degradation ability between both strains. This work highlights the lack of knowledge about the functional capacities of giant viruses and the imperative need to use computational tools to intend hypotheses that have to be tested experimentally at the bench.

## Data Availability Statement

Publicly available datasets were analyzed in this study (the genome of Tupanvirus soda lake and *Tupanvirus deep ocean*). This data can be found here: http://www.ncbi.nlm.nih.gov/genome under the accession number KY523104.1 and MF405918.1.

## Author Contributions

AL and BL designed and supervised the study. CR and AL performed the experiments and wrote the manuscript. All authors read and approved the final version of the manuscript.

## Conflict of Interest

The authors declare that the research was conducted in the absence of any commercial or financial relationships that could be construed as a potential conflict of interest.
