# Supplementary figures and images for: How Tupanvirus Degrades the Ribosomal RNA of Its Amoebal Host? The Ribonuclease T2 Track
Source: Front Microbiol. 2020 Jul 28;11:1691. doi: 10.3389/fmicb.2020.01691 (PMC7399046; doi:10.3389/fmicb.2020.01691)

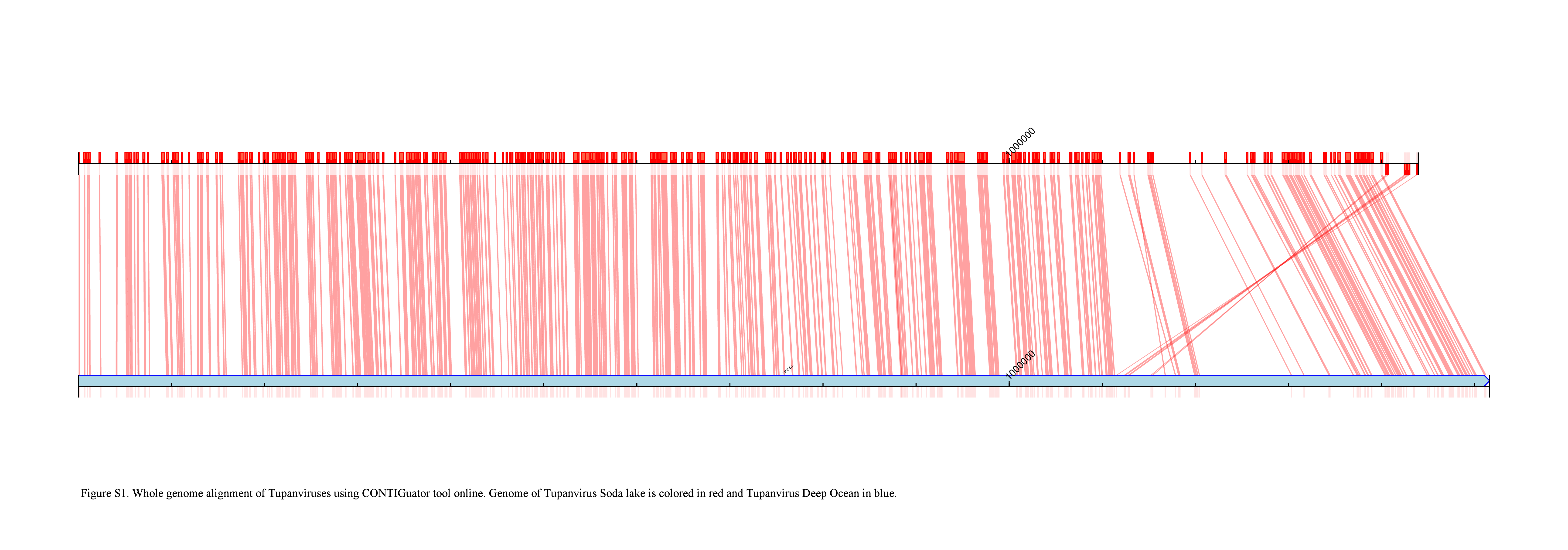

Supplement: Supplementary file 1 [file Image_1.TIFF]
