# Supplementary material for: How Tupanvirus Degrades the Ribosomal RNA of Its Amoebal Host? The Ribonuclease T2 Track
Source: Front Microbiol. 2020 Jul 28;11:1691. doi: 10.3389/fmicb.2020.01691 (PMC7399046; doi:10.3389/fmicb.2020.01691)
Supplement: Supplementary file 2 [file Table_1.docx]

| Predicted genes | Blastp results | CD-Search results | Accession |
| --- | --- | --- | --- |
| *Tupanvirus DEEP OCEAN* | | | |
| TPV-SL_51 | Putative ORFan | - | - |
| TPV-SL_82 | Putative ORFan | - | - |
| TPV-SL_101 | hypothetical protein | - | - |
| TPV-SL_106 | Putative ORFan | - | - |
| TPV-SL_113 | Putative ORFan | Nucleotydylyl transferase | cl00015 |
| TPV-SL_125 | Hypothetical protein | Beta_helix | pfam13229 |
| TPV-SL_126 | Putative HNH endonuclease | HNH endonuclease | cd00085 |
| TPV-SL_127 | Hypothetical protein | - | - |
| TPV-SL_156 | NAD dependent dehydratase | Epimerase | pfam01370 |
| TPV-SL_161 | Putative ORFan | - | - |
| TPV-SL_195 | NAD dependent DNA ligase | Helicase_C | pfam00271 |
| TPV-SL_240 | Putative ORFan | - | - |
| TPV-SL_259 | Putative ORFan | - | - |
| TPV-SL_285 | Hypothetical protein | - | - |
| TPV-SL_291 | Alkaline phosphatase | Phosphodiesterase/alkaline phosphatase D | COG3540 |
| TPV-SL_307 | Putative membrane protein | - | - |
| TPV-SL_338 | Hypothetical protein | Band_7 | pfam01145 |
| TPV-SL_339 | Hypothetical protein | Band_7 | pfam01145 |
| TPV-SL_361 | Transposase | DDE_3 | pfam13358 |
| TPV-SL_394 | Mg386 protein | - | - |
| TPV-SL_455 | Hypothetical protein | - | - |
| TPV-SL_456 | Hypothetical protein | - | - |
| TPV-SL_474 | Putative ORFan | - | - |
| TPV-SL_518 | Hypothetical protein | - | - |
| TPV-SL_543 | Hypothetical protein | Cysteine proteinase | Ssf54001 |
| TPV-SL_553 | Putative ORFan | - | - |
| TPV-SL_555 | Hypothetical protein | - | - |
| TPV-SL_569 | Hypothetical protein | - | - |
| TPV-SL_575 | Putative ORFan | - | - |
| TPV-SL_655 | Leucine rich repeat protein | Leucin rich repeat protein | COG4886 |
| TPV-SL_665 | Hypothetical protein | DUF2263 | pfam10021 |
| TPV-SL_671 | Putative ORFan | - | - |
| TPV-SL_720 | Putative ORFan | - | - |
| TPV-SL_731 | Putative ORFan | - | - |
| TPV-SL_740 | Hypothetical protein | - | - |
| TPV-SL_771 | Putative ORFan | - | - |
| TPV-SL_778 | Putative ORFan | - | - |
| TPV-SL_787 | Hypothetical protein | Myo-inositol oxygenase | pfam05153 |
| TPV-SL_896 | Putative HNH endonuclease | HNH endonuclease | Pfam13392 |
| TPV-SL_944 | putative orfan | - | - |
| TPV-SL_946 | T5orf172 domain containing protein | Zf_C2H2_type | Pfam00096 |
| TPV-SL_948 | hypothetical protein | - | - |
| TPV-SL_952 | EstA family serine hydrolase | Beta-lactamase | Pfam00144 |
| TPV-SL_953 | Endotype 6-aminohexoate-oligomer hydrolase | nyC-like family | cd02252 |
| TPV-SL_958 | Putative ORFan | - | - |
| TPV-SL_959 | Phosphatase II | Dual-specificity phosphatase domain | cd14498 |
| TPV-SL_961 | Hypothetical protein | - | - |
| TPV-SL_965 | Putative ORFan | - | - |
| TPV-SL_969 | Putative ORFan | - | - |
| TPV-SL_971 | hypothetical protein | C-term E3 ligase | cl26018 |
| TPV-SL_983 | Putative ORFan | - | - |
| TPV-SL_984 | Putative ORFan | - | - |
| TPV-SL_986 | Putative ORFan | - | - |
| TPV-SL_987 | Putative ORFan | - | - |
| TPV-SL_988 | Putative ORFan | - | - |
| TPV-SL_992 | Family 25 protein | Glycosyltransferase family 25 | Cd06532 |
| TPV-SL_1005 | Putative ORFan | - | - |
| TPV-SL_1020 | Hypothetical protein | - | - |
| TPV-SL_1028 | Putative ORFan | - | - |
| TPV-SL_1034 | Putative ORFan | - | - |
| TPV-SL_1036 | Putative ORFan | - | - |
| TPV-SL_1038 | Mg586 protein | Nucleotidyltransferase_POLXc | Cd00141 |
| TPV-SL_1048 | Putative ORFan | - | - |
| TPV-SL_1050 | CHDR domain protein | CHRD domain | Pfam07452 |
| TPV-SL_1052 | Putative ORFan | - | - |
| TPV-SL_1057 | Putative ORFan | - | - |
| TPV-SL_1097 | Putative ORFan | - | - |
| TPV-SL_1101 | Putative ORFan | - | - |
| TPV-SL_1108 | Putative ORFan | - | - |
| TPV-SL_1124 | Putative ORFan | - | - |
| TPV-SL_1177 | Putative ORFan | - | - |
| TPV-SL_1250 | Putative ORFan | T5orf172 | pfam10544 |
| *Tupanvirus SODA LAKE* | | | |
| TPV-SL_11 | hypothetical protein | - | - |
| TPV-SL_49 | putative orfan | - | - |
| TPV-SL_50 | Copine | vWA_copine-like | cd01459 |
| TPV-SL_51 | hypothetical protein | Phytanoyl-CoA dioxygenase | pfam05721 |
| TPV-SL_74 | putative orfan | - | - |
| TPV-SL_83 | Ankyrin | Ankyrin_2 | pfam12796 |
| TPV-SL_84 | Ankyrin repeat protein | Ankyrin repeat-containing domain | Ssf48403 |
| TPV-SL_90 | glycogen debranching enzyme alpha-1,6-glucosidase | Amylo-alpha-1,6-glucosidase | pfam06202 |
| TPV-SL_100 | putative orfan | - | - |
| TPV-SL_130 | putative orfan | - | - |
| TPV-SL_140 | putative orfan | - | - |
| TPV-SL_141 | putative orfan | - | - |
| TPV-SL_142 | putative orfan | - | - |
| TPV-SL_143 | putative orfan | - | - |
| TPV-SL_144 | putative orfan | - | - |
| TPV-SL_145 | putative orfan | - | - |
| TPV-SL_161 | Zn-dependent alcohol dehydrogenase | Cinnamyl alcohol dehydrogenase | cd05283 |
| TPV-SL_178 | putative orfan | - | - |
| TPV-SL_183 | putative orfan | - | - |
| TPV-SL_201 | methyltransferase FkbM family protein | methyltransferase, FkbM family | TIGR01444 |
| TPV-SL_242 | putative orfan | - | - |
| TPV-SL_243 | putative orfan | - | - |
| TPV-SL_253 | I cysteine dioxygenase | Cysteine dioxygenase type I | pfam05995 |
| TPV-SL_255 | Hypothetical protein | - | - |
| TPV-SL_256 | putative orfan | - | - |
| TPV-SL_280 | Hypothetical protein | - | - |
| TPV-SL_286 | monooxygenase | 2-polyprenyl-6-methoxyphenol hydroxylase and related FAD-dependent oxidoreductase family | COG0654 |
| TPV-SL_292 | hypothetical protein | - | - |
| TPV-SL_295 | putative orfan | - | - |
| TPV-SL_312 | putative orfan | - | - |
| TPV-SL_330 | T2 family ribonuclease | RNase_T2_euk | cd01061 |
| TPV-SL_350 | triacylglycerol lipase | Abhydrolase_3 | pfam07859 |
| TPV-SL_351 | putative alpha/beta hydrolase | Acetyl esterase/lipase | COG0657 |
| TPV-SL_374 | putative orfan | - | - |
| TPV-SL_388 | putative orfan | - | - |
| TPV-SL_391 | putative orfan | - | - |
| TPV-SL_392 | putative orfan | - | - |
| TPV-SL_404 | putative orfan | - | - |
| TPV-SL_413 | putative orfan | - | - |
| TPV-SL_477 | hypothetical protein | - | - |
| TPV-SL_491 | hypothetical protein | - | - |
| TPV-SL_500 | hypothetical protein | O-acetyl-ADP-ribose deacetylase | COG2110 |
| TPV-SL_506 | putative orfan | - | - |
| TPV-SL_545 | hypothetical protein | S-adenosylmethionine-dependent methyltransferase | cd02440 |
| TPV-SL_557 | hypothetical protein | - | - |
| TPV-SL_560 | hypothetical protein | - | - |
| TPV-SL_593 | hypothetical protein | - | - |
| TPV-SL_600 | putative orfan | - | - |
| TPV-SL_601 | putative orfan | - | - |
| TPV-SL_613 | hypothetical protein | - | - |
| TPV-SL_615 | putative orfan | - | - |
| TPV-SL_617 | hypothetical protein | Ankyrin_2 | pfam12796 |
| TPV-SL_618 | DnaJ-like subfamily C member 3 | DnaJ | cd06257 |
| TPV-SL_626 | putative orfan | - | - |
| TPV-SL_644 | putative orfan | - | - |
| TPV-SL_670 | putative orfan | - | - |
| TPV-SL_678 | dynamin family protein | Dynamin like-protein_1 | cd08771 |
| TPV-SL_682 | putative orfan | - | - |
| TPV-SL_699 | hypothetical protein | - | - |
| TPV-SL_717 | putative orfan | - | - |
| TPV-SL_724 | hypothetical protein | - | - |
| TPV-SL_822 | putative orfan | - | - |
| TPV-SL_847 | putative orfan | - | - |
| TPV-SL_850 | Aldehyde activiting protein | - | - |
| TPV-SL_883 | hypothetical protein | - | - |
| TPV-SL_923 | hypothetical protein | Nudix hydrolase 18 | cd04677 |
| TPV-SL_963 | putative orfan | - | - |
| TPV-SL_1008 | alcohol deshydrogenase | Cinnamyl alcohol dehydrogenase | cd05283 |
| TPV-SL_1011 | hypothetical protein | Glyco_tranf_GTA_type | cd00761 |
| TPV-SL_1021 | hypothetical protein | - | - |
| TPV-SL_1023 | putative orfan | - | - |
| TPV-SL_1025 | putative orfan | - | - |
| TPV-SL_1026 | putative orfan | - | - |
| TPV-SL_1040 | putative orfan | - | - |
| TPV-SL_1041 | putative orfan | - | - |
| TPV-SL_1042 | zf-C2H2-N-terminal protein | zf-C2H2 | pfam00096 |
| TPV-SL_1043 | hypothetical protein | - | - |
| TPV-SL_1044 | dolichyldiphosphatase | PAP2_dolichyldiphosphatase | cd03382 |
| TPV-SL_1054 | Hypothetical protein | - | - |
| TPV-SL_1074 | putative orfan | - | - |
| TPV-SL_1075 | putative orfan | - | - |
| TPV-SL_1076 | F-box domain protein | AMN1 domain | cd09293 |
| TPV-SL_1077 | S-phase kinase-associated protein 2 | F-box domain | pfam00646 |
| TPV-SL_1078 | hypothetical protein | F-box domain | smart00256 |
| TPV-SL_1079 | hypothetical protein | F-box domain | smart00256 |
| TPV-SL_1080 | F-box domain protein | AMN1 domain | cd09293 |
| TPV-SL_1081 | hypothetical protein | F-box domain | smart00256 |
| TPV-SL_1084 | putative orfan | Thioredoxin reductase | COG0492 |
| TPV-SL_1085 | hypothetical protein | - | - |
| TPV-SL_1086 | cytochrome P450 CYP736A12-like protein | p450 | pfam00067 |
| TPV-SL_1087 | putative orfan | - | - |
| TPV-SL_1092 | putative ankyrin repeat protein | Ankyrin_2 | pfam12796 |
| TPV-SL_1094 | putative ankyrin repeat protein | Ankyrin_2 | pfam12796 |
| TPV-SL_1095 | ankyrin repeat protein | Ankyrin_2 | pfam12796 |
| TPV-SL_1096 | putative orfan | - | - |
| TPV-SL_1097 | hypothetical protein | - | - |
| TPV-SL_1098 | hypothetical protein | - | - |
| TPV-SL_1099 | putative sérine/threonine protein kinase | PKc_MKK7 | cd06618 |
| TPV-SL_1100 | putative orfan | - | - |
| TPV-SL_1101 | hypothetical protein | phosphodiesterase | PKR12704 |
| TPV-SL_1102 | putative orfan | - | - |
| TPV-SL_1104 | putative orfan | - | - |
| TPV-SL_1105 | hypothetical protein | - | - |
| TPV-SL_1106 | putative orfan | - | - |
| TPV-SL_1108 | hypothetical protein | Ras_like_GTPase | cd00882 |
| TPV-SL_1109 | hypothetical protein | - | - |
| TPV-SL_1110 | putative orfan | - | - |
| TPV-SL_1112 | putative orfan | - | - |
| TPV-SL_1114 | hypothetical protein | - | - |
| TPV-SL_1115 | hypothetical protein | - | - |
| TPV-SL_1117 | hypothetical protein | - | - |
| TPV-SL_1118 | hypothetical protein | - | - |
| TPV-SL_1119 | putative orfan | - | - |
| TPV-SL_1121 | putative orfan | - | - |
| TPV-SL_1123 | putative orfan | - | - |
| TPV-SL_1126 | hypothetical protein | - | - |
| TPV-SL_1129 | putative orfan | - | - |
| TPV-SL_1140 | putative orfan | - | - |
| TPV-SL_1142 | linear amide C-N hydrolase | Penicillin V acylase | cd00542 |
| TPV-SL_1143 | CLUMA CG011169 isoform A | RFX_DNA_binding | pfam02257 |
| TPV-SL_1144 | putative orfan | - | - |
| TPV-SL_1145 | hypothetical protein | CAP_euk | cd05380 |
| TPV-SL_1151 | Putative orfan | - | - |
| TPV-SL_1156 | putative orfan | - | - |
| TPV-SL_1157 | Nad dependent amine oxidase | Amino_oxidase | pfam01593 |
| TPV-SL_1158 | putative orfan | - | - |
| TPV-SL_1159 | putative orfan | - | - |
| TPV-SL_1160 | glucose-methanol-choline oxidoreductase | Choline deshydrogenase | COG2303 |
| TPV-SL_1163 | putative orfan | - | - |
| TPV-SL_1172 | collagen like protein | Collagen | pfam01391 |
| TPV-SL_1173 | putative orfan | - | - |
| TPV-SL_1178 | hypothetical protein | Exonuclease/endonuclease/ phosphatase EEP_1 | cd09083 |
| TPV-SL_1185 | Putative orfan | - | - |
| TPV-SL_1209 | 1-deoxy-D-xylulose-phosphate synthase | Thiamine pyrophosphate family, Transketolase (TPP_TK) | cd02012 |
| TPV-SL_1226 | putative orfan | - | - |
| TPV-SL_1228 | hypothetical protein | Peptidase_S74 | pfam13884 |
| TPV-SL_1253 | hypothetical protein | - | - |
| TPV-SL_1255 | hypothetical protein | Mth938 domain | cd05126 |
| TPV-SL_1267 | hypothetical protein | Ankyrin_2 | pfam12796 |
| TPV-SL_1289 | Putative orfan | - | - |
| TPV-SL_1294 | glycosyltransferase | Glyco_transf_GTA_type | cl11394 |
| TPV-SL_1365 | Putative orfan | - | - |
| TPV-SL_1371 | Putative orfan | - | - |

**Supplementary Table 1.** List of unique proteins from Tupanvirus Soda Lake and Tupanvirus Deep Ocean. Results corresponded to analyses with Blastp and CD-Search tools online (threshold e-value > 0,001 for both).
